# β-Cyclocitral-Mediated Metabolic Changes Optimize Growth and Defense Responses in *Solanum lycopersicum* L.

**DOI:** 10.3390/metabo13030329

**Published:** 2023-02-23

**Authors:** Shreyas Deshpande, Sirsha Mitra

**Affiliations:** Department of Botany, Savitribai Phule Pune University, Pune 411007, India

**Keywords:** apocarotenoids, herbivory, liquid chromatography-mass spectrometry, metabolomics, stress signaling

## Abstract

β-cyclocitral (βCC) is one of the significant oxidative products of β-carotene. It primes plants for multiple stress acclimation without compromising plant growth. Metabolic reorganization is necessary to maintain a balance between growth and defense. However, the βCC-mediated changes in a plant’s metabolic network are unknown. Here, we demonstrate how βCC-induced metabolic changes enable *Solanum lycopersicum* L. (tomato) plants to promote defense and maintain growth under stress. An analysis of early (0–240 min) and late (72 h) changes in the tomato metabolome after βCC-treatment using liquid chromatography and tandem mass spectrometry identified 57 compounds. A principal coordinate analysis suggested that βCC treatment significantly changes the metabolite profile. A variable importance in projection (VIP) analysis revealed 16 and 19 discriminant metabolites from early and late samples, respectively (VIP ≥ 1.0). Upregulated metabolites were mainly amino acids and phytophenols. Pathway enrichment analysis showed that βCC treatment influenced amino acid metabolism at early and later times; however, phenylpropanoid and isoquinoline biosynthesis were influenced only at the later time. A 66.6% similarity in the upregulated metabolites of βCC- and simulated-herbivory-treated plants confirmed βCC’s role against herbivores. We conclude that βCC steers a temporal separation in amino acids and defense metabolite accumulation that optimizes resource allocation to growth and defense.

## 1. Introduction

Environmental stresses are deleterious for the survival of plants. *Solanum lycopersicum* L. (tomato), one of the major food crops in the family Solanaceae, is also challenged by multiple environmental stresses, namely fungal pathogens, herbivores, cold, drought, etc. [1,2]. To counteract environmental challenges, plants optimize their defense strategies via stress sensing, signaling, and reorganizing their metabolic profile under the environmental stimuli. The chloroplast plays a central role in plant defense signaling as it harbors biosynthetic pathways of significant defense metabolites; chloroplast-associated carotenoids are crucial for protecting the photosynthetic apparatus from photo-oxidative damage [3] because the presence of a C40 polyene backbone makes carotenoids susceptible to oxidative cleavage. Cleavage of carotenoids occurs enzymatically by carotenoid cleavage dioxygenase or non-enzymatically by reactive oxygen species (ROS). The oxidative carbonyl products of carotenoids are known as apocarotenoids [4,5]. Apocarotenoids are well known as the precursors of abscisic acid, a phytohormone involved in abiotic stress responses [6]. In recent years, the identification of novel apocarotenoids has established their role in stress signaling [5,7]. Previous studies showed that singlet oxygen (^1^O_2_)-mediated cleavage of β-carotene produces many apocarotenoids, where β-cyclocitral (βCC) is the major apocarotenoid [8].

Interestingly, drought, photooxidative stress, and herbivory increase the level of βCC [9,10,11,12]. Studies have shown that exogenous application of βCC can induce ^1^O_2_-responsive genes, marker genes for photo-oxidative stress [8]. Recently, we found that βCC treatment triggers the accumulation of transcripts related to both abiotic and biotic stresses. Our study also revealed that exogenous application of βCC primes plants against drought and develops resistance against herbivory [11,13]. These results suggest that βCC causes functional changes in addition to trancriptomic changes. On the other hand, βCC treatment enhances the growth of primary roots and the branching of lateral roots [11]. As defenses are costly, a trade-off between growth and defense is often evident in stress-exposed plants. However, βCC treatment enhances both plant growth and defense. Metabolites can influence plant growth, the branching pattern of roots and shoots, the size, shape, and position of leaves, etc. [14,15,16]. Therefore, metabolic reorganization is a prerequisite to maintaining a balance between growth and defense. However, βCC-mediated changes in the plant metabolome are yet to be revealed.

In the ‘omics’ field, metabolomics identifies large-scale changes in the plant metabolome. It is highly challenging, as plant metabolites possess different physical and chemical properties and vary significantly in their concentrations [17]. Metabolomics can be investigated using two main approaches: targeted and untargeted [18,19]. Initially, chromatographic techniques, such as liquid chromatography (LC) and gas chromatography (GC), separate the metabolites, and mass spectroscopy (MS) investigates the quantitative changes in the plant metabolome [20]. The use of nuclear magnetic resonance (NMR) spectroscopy is also evident in metabolomics analysis [21]; however, as the sensitivity of MS is high, it is preferred over NMR spectroscopy for analyzing plant metabolites [22]. The tomato has been considered a model fruit. Its metabolomic profiles have been studied to discriminate different varieties, determine geographical origin, examine fruit development and ripening, and investigate seasonal changes [23]. A comparative metabolomics study of *Ralstonia solanacearum*-infected tomato leaves, stems, and roots revealed the metabolic changes after being infected with *R. solanacearum* [24]. This study identified the metabolites providing plant defense against *R. solanacearum* [24]. However, a comparative metabolomics study of tomato plants after apocarotenoid treatment has yet to be executed. 

In light of the reported results and previous findings, we hypothesized that βCC treatment reorganizes plants’ metabolic networks to enhance plant growth and defense. Therefore, in the current study, we aimed to reveal how exogenous application of βCC changes the metabolic signature of the tomato plants that enables them to maintain growth under stress using an LC–MS-based approach.

## 2. Materials and Methods

### 2.1. Plant Material, Growth Conditions, and Treatments

Seeds of tomato (var. Pusa Ruby) were germinated on cocopeat. 15-day-old seedlings were transferred in pots (9 cm × 9 cm) that contained soil, cocopeat, and vermiculite (5:4:1) and raised for five weeks. These plants were treated with 1 mL of pure βCC (treatment), or water (control) kept in a watch glass in a transparent, closed glass container [11,25]. Tissues were harvested in liquid nitrogen 0, 30, 60, 90, 180, 240 min, and 72 h after treatment. Insect herbivory was simulated in tomato plants by wounding the tomato leaves with a fabric pattern wheel parallel to the mid-vein and applying 20 µL of oral secretion of *Spodoptera litura* larvae diluted with sterile water (1:1) to the wound [26]. Tissues were harvested in liquid nitrogen 72 h after treatment. All harvested tissues were stored at −80°C until further use. 

### 2.2. Extraction of Metabolites and Data Acquisition in LC-QTOF-MS/MS 

Leaf samples of tomato plants were pulverized in liquid nitrogen. An amount of 200 mg of tissue was extracted with 1 mL methanol spiked with the internal standard formononetin (10 µg ml^−1^) by vortexing continuously for 15 min. Further, the extracts were centrifuged at 15,000× *g* at 4 °C for 20 min. The collected supernatant was filtered and subjected to LC-QTOF-MS/MS (Agilent Technologies, Stuttgart, Germany) for analysis. 

The extracts were separated on an XDB-C18 column (150 mm × 4.6 mm × 5 µm; Agilent Zorbax-Eclipse) using 0.1% (*v*/*v*) formic acid (solvent A) and acetonitrile with 0.1% formic acid (solvent B) as mobile phases. Compounds were eluted with a solvent gradient profile consisting of 95% A for 1 min followed by a gradient that reached 95% B by 15 min, returned 95% A by 17.5 min, and continued until 20 min. The injection volume was adjusted to 10 µL. The eluted compounds were detected using the centroid mode for both negative and positive ionization modes. The pump limit was 1 min; the draw speed and eject speed were set to 200 µL min^−1^ and 400 µL min^−1^, respectively. The maximum pressure limit in the column was 800 bar, and the retention time exclusion tolerance was set to (±) 0.2 min. The ion source (dual ESI) was adjusted with a limit of 2 precursors min^−1^.

### 2.3. LC-QTOF-MS/MS Data Analysis 

Initially, a personal compound database library (PCDL) was prepared by accessing different databases and libraries for the metabolites from Solanaceae plants. The PCDL library and the Mass Hunter Qualitative Analysis BO.07.00 tool (Agilent Technologies) were used to analyze the spectra of the metabolites. The MS/MS data were procured from four replicate samples to assess the biological variations in the control, βCC-, and simulated-herbivory-treated samples. Compounds with an abundance greater than 10,000 counts and a score of more than 75 were considered for further analysis. These compounds were identified with a mass threshold of 7 ppm and a peak distance threshold of 10 ppm in MS/MS mode using the ‘find by formula’ function in the Mass Hunter software. The molecular ion and daughter ions in MS and MS/MS modes were compared with the reference or predicted spectra available at the Human Metabolome Database (HMDB), MassBank, and Pubchem.

### 2.4. Statistical Analysis

Principal coordinate analysis (PCoA) of metabolomics data was performed in PAST 3 [27]. A heatmap was prepared with the web tool ClustVis (https://biit.cs.ut.ee/clustvis/; accessed on 1 December 2022). Metaboanalyst 5.0 (https://www.metaboanalyst.ca/; accessed on 1 December 2022) was used for orthogonal partial least square discriminant analysis (OPLS-DA), variable importance in projection (VIP), fold-change analysis, and pathway analysis. Venn diagrams were created using Venny 2.1 (https://bioinfogp.cnb.csic.es/tools/venny/; accessed on 1 December 2022) [28]. Normalized peak areas were analyzed by one-way ANOVA followed by Fisher’s least significant difference (LSD) post hoc test. The significance was determined at *p* ≤ 0.05.

## 3. Results

### 3.1. Application of βCC Altered the Metabolite Profile of Tomato Plants 

**Figure 1:**
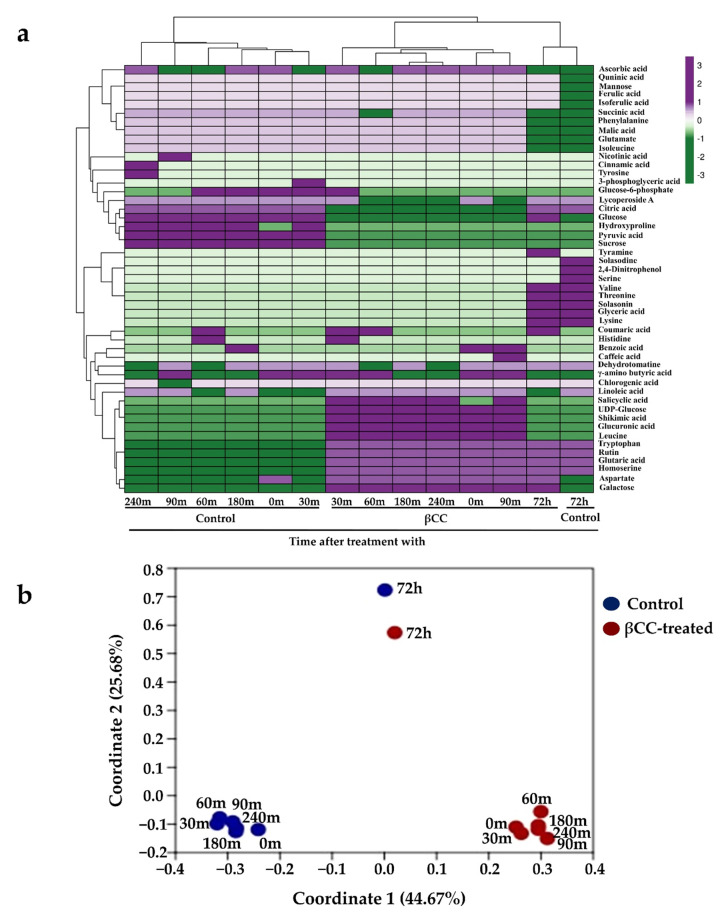
Variation in the metabolites after βCC treatment across different time points. (**a**) The metabolites showed variation in their accumulation pattern across different time points after being treated with βCC. The color scheme at the top-right corner codes for the z-scores (−3 to +3) calculated over binary coordinates of the samples. The average linkage clustering computed between the samples is based on the Manhattan distance and depicted at the top of the heatmap. (**b**) The principal coordinate analysis (PCoA) was performed with the identified metabolites using the Jaccard coefficient and transformation exponent value 2. It showed that the maximum percentage of the variation was captured in coordinates 1 and 2. The values in parentheses show the percentages of the variation.

Previous reports showed that exogenous βCC could improve plants’ tolerance to different stresses but did not reduce plant growth [9,11]. Therefore, to investigate how βCC improves plants’ tolerance without compromising plant growth, we analyzed the metabolome of tomato plants after βCC treatment and compared it with control plants. Samples were collected at 0, 30, 60, 90, 180, and 240 min after βCC treatment to assess βCC-mediated early changes in the metabolome (Figure S1). Similarly, to track the βCC-induced late metabolic changes, samples were collected at 72 h after βCC treatment (Figure S1). A total of 57 compounds were identified from positive and negative modes across all the time points. Compounds were identified based on the daughter ion spectra at three energy levels 10 eV, 20 eV, and 40 eV (Table S1). Among the identified compounds, 45 and 31 were accumulated at early (0–240 min) and late (72 h) time points, respectively. A heatmap created with the identified compounds showed that the metabolic profiles of βCC-treated samples differed from that of the control (Figure 1a). Principal coordinate analysis (PCoA) showed a total variability of 70.35%, where the maximum variation was captured in coordinates 1 (44.67%) and 2 (25.68%). Interestingly, βCC-treated samples from 0–240 min were grouped separately from the control samples from the same time points (0–240 min). However, the control and βCC-treated samples from 72 h were grouped in the same coordinate in the PCoA plot (Figure 1b). This suggests that βCC-mediated early changes in the metabolite profile were more prominent than later changes. 

### 3.2. Regulation of Metabolites in Early Time Points after βCC Treatment 

**Figure 2:**
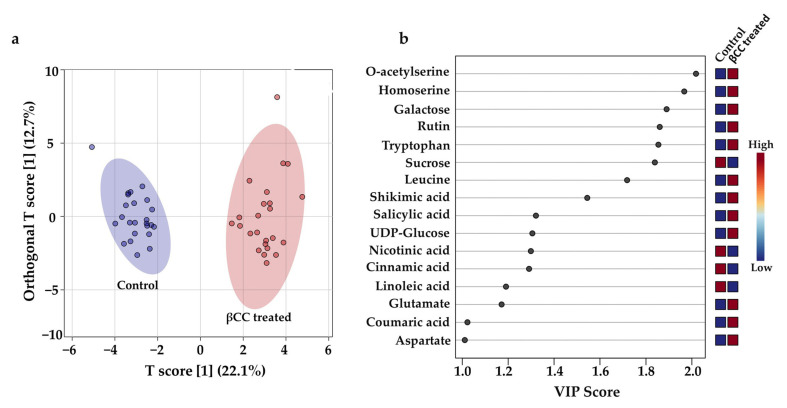
βCC-induced early metabolic changes and discriminant metabolites. To examine the differences in the metabolic profile in tomato plants early after βCC treatment, orthogonal partial least square discriminant analysis (OPLS-DA) and variable importance in projection (VIP) analysis were performed with the normalized peak area of the metabolites identified from 0–240 min after βCC treatment and in control plants. (**a**) An OPLS-DA plot showed the control group clustered to the left region and the βCC-treated group clustered to the right area in the OPLS-DA score plot. The shaded ellipses represent the confidence interval of 95% from OPLS-DA models. (**b**) A variable importance in projection (VIP) plot showed that the metabolites responsible for the significant separation observed between the two sample groups were indicated by a VIP score ≥ 1.0. An increase in VIP score indicates a high contribution of the metabolites to the group separation. The red and blue boxes on the right indicate whether the metabolite concentration was high (red) or low (blue) in the βCC-treated plants compared to control plants.

The PCoA plot indicated a pronounced effect of βCC on metabolite accumulation within 0–240 min after the treatment. To find out the discriminant metabolites that were responsible for separating the control and βCC-treated samples, the peak area of 45 identified compounds from 0–240 min time scale were normalized with the internal standard and analyzed for an orthogonal partial least square discriminant analysis (OPLS-DA) and variable importance in projection (VIP) analysis. OPLS-DA showed that the control and βCC-treated samples were well separated in the score plot (Figure 2a). The data confirmed that the metabolic blend of the control and βCC-treated samples were different. VIP analysis determined the relative contribution of the metabolites to the variation between the control and βCC-treated samples. The analysis identified a total of 16 discriminant compounds (VIP score ≥ 1.0) (Figure 2b).

**Figure 3:**
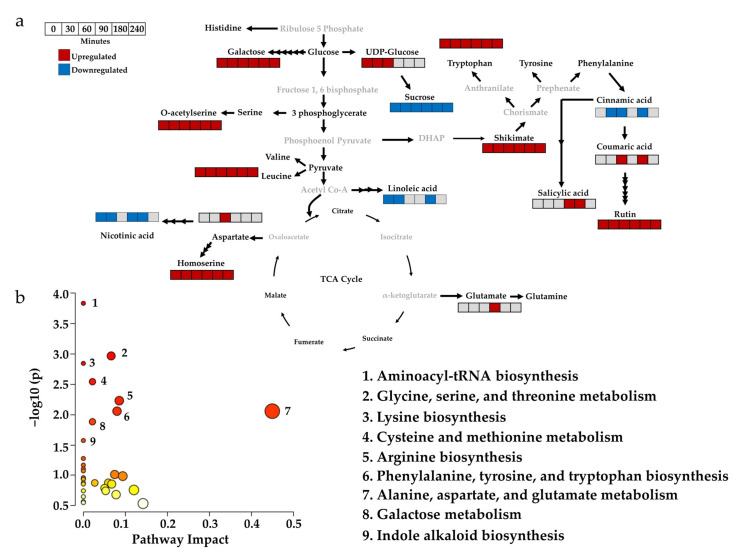
Mapping the relative expression of discriminant metabolites early after βCC treatment and their influence on metabolic pathways. The normalized peak area of the discriminant metabolites from control and βCC-treated samples of different time points (0, 30, 60, 90, 180, and 240 min) were subjected to one-way ANOVA; the significance was determined by Fisher’s LSD at *p* = 0.05. (**a**) Significantly up- and down-regulated metabolites from different time points are mapped on the pathways modified from the KEGG database. (**b**) Pathway analysis using the upregulated discriminant metabolites shows altered pathways after βCC treatment (left panel). The x-axis depicts pathway impact values obtained from the pathway topology analysis, and the y-axis depicts the –log of the *p* values obtained from the pathway enrichment analysis (*p* ≤ 0.05). Circular nodes represent the metabolic pathways. The size of the circular nodes positively corresponds with the impact of the proposed pathway based on the pathway topology. The node color, from yellow to orange, shows different levels of significance based on pathway enrichment analysis (yellow–low; orange–high). The most significantly altered pathways are characterized by both a high −log(*p*) value and a high impact value. Nine pathways were significantly altered after βCC treatment (right panel). Metabolites in black font were identified in the study, but greys were not. Grey boxes indicate no significant changes in the metabolite accumulation at *p*≤ 0.05.

Further, the fold-change analysis revealed that, out of 16 discriminant compounds, 11 were upregulated (log_2_FC ≥ 2), and four were downregulated (log_2_FC ≤ −2.0) (Figure S2). Though the log_2_FC value of one of the discriminant metabolites (aspartate) was 1.8, its VIP score was 1.01; therefore, it had the most negligible influence in separating the control and βCC-treated sample groups. The normalized peak area of the discriminant metabolites from all the time points was further analyzed by one-way ANOVA and Fisher’s LSD post hoc test. Significantly different metabolites were determined at *p* ≤ 0.05 (Table S2) and visualized by mapping their regulation in the network of metabolic pathways for each time point (Figure 3a). The analysis showed that the upregulated metabolites were mainly amino acids and their derivatives (i.e., tryptophan, leucine, aspartate, glutamate, homoserine, and o-acetylserine), followed by phytophenols (i.e., shikimate, rutin, and coumaric acid), carbohydrates and their derivatives (i.e., UDP-glucose and galactose), and carboxylic acid (i.e., salicylic acid). Cinnamic acid, linoleic acid, nicotinic acid, and sucrose were significantly downregulated metabolites. Most of the compounds were upregulated, so a pathway enrichment analysis was performed with the upregulated compounds. It revealed that the pathways related to (1) aminoacyl-tRNA biosynthesis (*p* = 0.0001), (2) glycine, serine, and threonine metabolism (*p* = 0.001), (3) lysine biosynthesis (*p* = 0.0010), (4) cysteine and methionine metabolism (*p* = 0.0028), (5) arginine biosynthesis (*p* = 0.0059) (6) phenylalanine, tyrosine, and tryptophan biosynthesis (*p* = 0.0088), (7) alanine, aspartate, and glutamate metabolism (*p* = 0.0088) (8) galactose metabolism (*p* = 0.0131), and (9) indole alkaloid biosynthesis (*p* = 0.0267) were significantly influenced (hypergeometric test; *p* ≤ 0.05) by βCC application (Figure 3b). The above data suggest that βCC treatment mainly boosted plants’ amino acid metabolism within a few hours of treatment.

### 3.3. Regulation of Metabolites at a Late Time Point after βCC Treatment 

**Figure 4:**
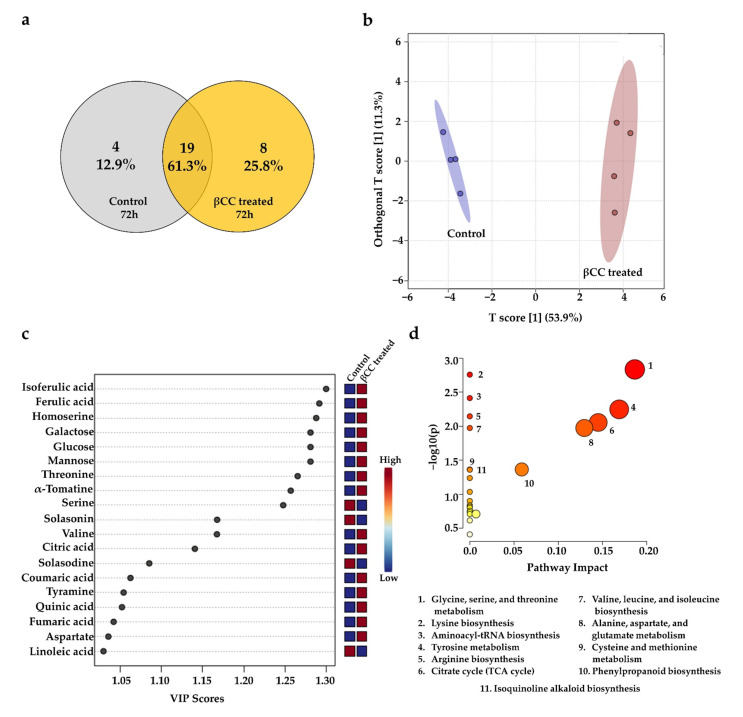
βCC-induced late metabolic changes, discriminant metabolites, and their influence on metabolic pathways. (**a**) A Venn diagram created with the identified metabolites from control and βCC-treated samples showed many metabolites regulated commonly and specifically in them. (**b**) OPLS-DA plot showed distinct clusters of the control group to the left region and the βCC-treated group to the right region of the plot. The shaded ellipses represent the confidence interval of 95% from OPLS-DA models. (**c**) A variable importance in projection (VIP) plot showed that the metabolites responsible for the significant separation observed between these two sample groups were indicated by a VIP score ≥ 1.0. An increase in VIP score indicates a high contribution of the metabolites to the group separation. The red and blue boxes on the right indicate whether the metabolite concentration was high (red) or low (blue) in the βCC-treated plants compared to control plants. (**d**) Pathway analysis using the upregulated discriminant metabolites shows altered pathways after βCC treatment (upper panel). The x-axis depicts pathway impact values obtained from the pathway topology analysis, and the y-axis depicts the –log of the *p* values obtained from the pathway enrichment analysis (*p* ≤ 0.05). Circular nodes represent the metabolic pathways. The size of the circular nodes positively corresponds with the impact of the proposed pathway based on the pathway topology. The node color, from yellow to orange, shows different levels of significance based on pathway enrichment analysis (yellow–low; orange–high). The most significantly altered pathways were characterized by both a high −log(*p*) value and a high impact value. A total of 11 pathways were significantly altered after βCC treatment (lower panel).

Generally, in plants, accumulation of defense metabolites takes place 3–4 days after exposure to stress [29]. Surprisingly, the PCoA plot indicated that the metabolic blend of βCC-treated samples from 72 h was less different than that of the control samples. This result suggests that quantitative changes were more critical than qualitative changes in later times. The Venn diagram constructed with the metabolites detected 72 h after βCC treatment showed that, among 31 identified compounds, 19 were commonly present (63%) in control and βCC-treated plants, and only eight compounds (25%) were detected explicitly after βCC treatment (Figure 4a). However, the OPLS-DA (Figure 4b) and fold-change analysis (Figure S3) showed that, though the metabolite profile of control and βCC-treated plants were qualitatively similar, they were quantitatively different. Further, we identified the discriminant metabolites from βCC-treated samples after 72 h of treatment by VIP analysis. This revealed 19 discriminant compounds, of which 15 compounds (78%) were significantly upregulated (log2FC ≥ 2.0), and four compounds (21%) were significantly downregulated (log2FC ≤ −2.0). The upregulated compounds included mainly phytophenols (i.e., isoferulic acid, ferulic acid, coumaric acid, and quinic acid), followed by amino acids (i.e., homoserine, threonine, valine, tyramine, and aspartate), carbohydrates and their derivatives (i.e., galactose, glucose, and mannose), organic acids (i.e., citric acid and fumaric acid), and steroidal alkaloids (i.e., α-tomatine) (Figure 4c). Significantly downregulated metabolites were serine, solasodine, solasonin, and linoleic acid (Figure 4c). The pathway enrichment analysis of the upregulated discriminant compounds revealed their involvement in (1) glycine, serine, and threonine metabolism (*p* = 0.0014), (2) lysine biosynthesis (*p* = 0.0017), (3) aminoacyl-tRNA biosynthesis (*p* = 0.0038), (4) tyrosine metabolism (*p* = 0.0056), (5) arginine biosynthesis (*p* = 0.0071), (6) citrate cycle (*p* = 0.0088), (7) valine, leucine and isoleucine biosynthesis (*p* = 0.0106), (8) alanine, aspartate and glutamate metabolism (*p* = 0.0106), (9) cysteine and methionine metabolism (*p* = 0.0433), (10) phenylpropanoid biosynthesis (*p* = 0.0433), and (11) isoquinoline alkaloid biosynthesis (*p* = 0.0437) (hypergeometric test; *p* ≤ 0.05) (Figure 4d). The data suggest that βCC treatment enhanced the pathways related to plant defense at later time points, keeping the amino acid biosynthesis and metabolism boosted. 

### 3.4. βCC Treatment Induces a Similar Metabolic Response as Simulated Herbivory

**Figure 5:**
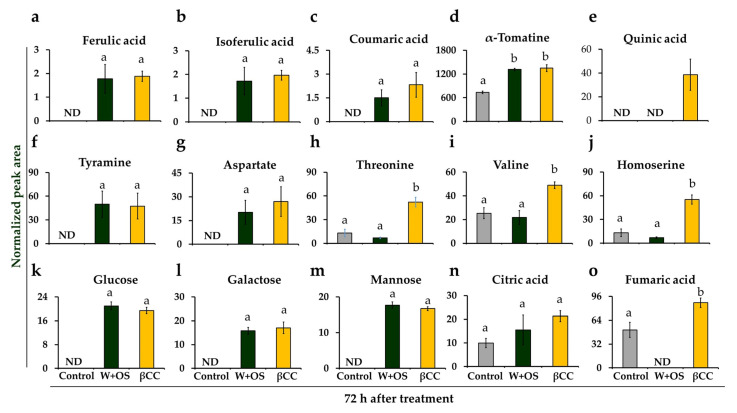
Comparative levels of metabolites after βCC treatment and simulated herbivory. The normalized peak area of the upregulated discriminant metabolites identified after 72 h of βCC treatment was compared with that of control and simulated-herbivory-treated (W+OS) plants. The accumulation of phytophenols, (**a**) ferulic acid (F_2,9_ = 7.975; *p*_βCC_ = 0.006, *p*_W+OS_ = 0.008), (**b**) isoferulic acid (F_2,9_ = 9.012; *p*_βCC_ = 0.0036, *p*_W+OS_ = 0.0078), (**c**) coumaric acid (F_2,9_ = 5.554; *p*_βCC_ = 0.014, *p*_W+OS_ = 0.081), and (**d**) α-tomatine (F_2,9_ = 37.185; *p*_βCC and W+OS_ < 0.001) were significantly more in βCC- and simulated herbivory-treated plants, but (**e**) quinic acid (F_2,9_ = 8.672; *p*_βCC_ = 0.0057, *p*_W+OS_ = NA) was upregulated only after βCC treatment. Similarly, the levels of amino acids, (**f**) tyramine was also upregulated in βCC- and simulated-herbivory-treated plants (F_2,9_ = 4.368; *p*_βCC_ = 0.034, *p*_W+OS_ = 0.027); however, levels of (**g**) aspartate (F_2,9_ = 4.082; *p*_βCC_ = 0.022, *p*_W+OS_ = 0.069), (**h**) threonine (F_2,9_ = 28.458; *p*_βCC_ = 0.0002, *p*_W+OS_ = 0.364), (**i**) valine (F_2,9_ = 10.038; *p*_βCC_ = 0.005, *p*_W+OS_ = 0.599), and (**j**) homoserine (F_2,9_ = 35.912; *p*_βCC_ < 0.001, *p*_W+OS_ = 0.341) were upregulated only after βCC treatment. Accumulation of carbohydrates and their derivatives, (**k**) glucose (F_2,9_ = 137.907; *p*_βCC and W+OS_ < 0.001), (**l**) galactose (F_2,9_ = 33.337; *p*_βCC and W+OS_ < 0.001), and (**m**) mannose (F_2,9_ = 260.128; *p*_βCC_ = 0.014, *p*_βCC and W+OS_ < 0.001) were also upregulated after both βCC treatment and simulated herbivory; however, accumulation of organic acids (**n**) citric acid (F_2,9_ = 1.980; *p*_βCC_ = 0.077, *p*_W+OS_ = 0.356), and (**o**) fumaric acid (F_2,9_ = 40.004; *p*_βCC_ = 0.005, *p*_W+OS_ = 0.0006) was only increased after βCC treatment. The mean normalized peak area (±SE) was analyzed from four replicate plants by one-way ANOVA and Fisher’s LSD post hoc test. Different letters indicate significant differences at *p* ≤ 0.05.

Previously, we found that βCC treatment was also able to enhance resistance against a generalist herbivore, *Spodoptera littoralis*, in *Arabidopsis thaliana* [13]. To investigate if the discriminant metabolites are also influenced after insect herbivory, we compared the levels of βCC-induced metabolites with simulated-herbivory-treated and control plants after 72 h of treatment. Interestingly, out of the 15 discriminant compounds from βCC-treated plants, ten compounds (66.6%) were significantly upregulated after simulated herbivory compared to the control (Figure 5). The upregulated compounds included ferulic acid, isoferulic acid, coumaric acid, α-tomatine, tyramine, aspartate, citric acid, galactose, glucose, and mannose (Figure 5). In addition, βCC-treated samples accumulated significantly more amounts of quinic acid, threonine, valine, homoserine, and fumaric acid (One-way ANOVA; Fisher’s LSD; *p* ≤ 0.05) (Figure 5). The data suggest that βCC treatment can induce a similar blend of compounds that are upregulated after insect herbivory with a few more amino acids and phytophenol. 

## 4. Discussion

Plant growth and productivity are severely affected by environmental stress. However, plants adapt to this unfavorable condition by optimizing their defense strategies. Therefore, the machinery of stress perception, signal transduction, and, ultimately, production of defense responses have been extensively studied. The plant hormones jasmonic acid [30], abscisic acid [31], ethylene [32], and salicylic acid [33], are known as stress signaling molecules; however, the growth hormones auxin, gibberellins, and strigolactones also participate as signaling molecules in plant–environment interactions [34]. In recent years researchers have uncovered apocarotenoids as potential signaling molecules. Studies have shown that βCC, the oxidative product of β-carotene, is a significant apocarotenoid that can induce ^1^O_2_-responsive genes, essential for photooxidative stress in *Arabidopsis thaliana* [8]. We found that the exogenous application of βCC reprograms the transcriptome of tomato plants [25] by triggering multiple stress-responsive genes, essential to counteract both abiotic and biotic stresses. We also found that βCC can prime tomato plants against drought and induce resistance against insect herbivores in *A. thaliana* plants [11,13]. However, βCC treatment does not negatively affect plant growth but enhances root growth and lateral branching [10,11]. Generally, plants compromise their photosynthetic ability under stress, and, at the same time, they invest in defense; this reduces the carbon flux towards growth and causes a growth–defense trade-off [35]. Previous studies on tomato metabolomics have revealed that the leaf, stem, and root metabolome present different signatures upon infestation. In addition, metabolomic markers can be used to monitor or predict the performance of plants and their response to environmental stresses [36]. These facts stimulated the hypothesis that βCC specifically upregulates those metabolites that play a dual role in improving plant growth and defense. It is also possible that defense metabolites are upregulated after accomplishing plant growth requirements in βCC-treated plants, which avoids diversion of carbon flux from growth toward defense. To examine this, we compared the metabolome of βCC-treated plants with control plants to reveal βCC-induced metabolic changes in tomato plants. In addition, we also compared βCC-induced metabolic changes with simulated-herbivory-induced changes to reveal βCC’s influence on the defense metabolites related to insect herbivory. Our results showed that βCC treatment mainly influenced the metabolism of amino acids and the accumulation of phytophenols.

Traditionally, amino acids are designated as the building blocks of protein; however, they also serve as intermediates for other biosynthetic pathways. Therefore, apart from plant growth and development, they also influence the generation of metabolic energy and signaling processes [37] and confer resistance to both abiotic and biotic stress [38,39,40]. In a recent study, it was demonstrated that pathogen-inoculated tomato plants’ primary metabolic pools were altered [41]. Similarly, βCC treatment induced many primary metabolites very early after the treatment. We found that βCC treatment increased the levels of tryptophan and its precursor shikimate. Shikimate is the common intermediate of the amino acid tryptophan and phenylalanine biosynthetic pathways. As tryptophan is the precursor for auxin and 5-hydroxytryptamine biosynthesis, an increase in the tryptophan level may contribute to plant growth. On the other hand, phenylalanine accumulation remained unaltered after βCC treatment; however, the derivatives of phenylalanine, namely, coumaric acid, rutin, and salicylic acid, were significantly increased (Figure 3a). These metabolites are known for their antioxidant and antipathogenic properties [42,43,44]. Moreover, rutin and coumaric acid application resulted in increased photosynthesis, chlorophyll content, and shoot growth [45,46]. In addition, the amino acids glutamate and glycine were also increased after βCC treatment. Glutamate is the precursor of chlorophyll tetrapyrrol protoporphyrin IX biosynthesis, where δ-aminolaevulinic acid (ALA) is the major intermediate [47]. Interestingly, the application of ^14^C-labeled glycine can instantly be incorporated into ALA in dark-grown barley leaves [48]. Moreover, exogenous glycine application can stimulate root hair formation and [49] induce plant growth [50,51]. These findings are consistent with our previous results that showed that βCC treatment could enhance chlorophyll accumulation and root growth in tomato plants [11].

βCC treatment can also enhance the levels of aspartate, which serves as a precursor of many biosynthetic pathways required for growth and defense [52]. For example, it is a precursor for the aspartate oxidase pathway that synthesizes nicotinamide adenine dinucleotide (NAD), an essential component of chlorophyll synthesis [53]. Therefore, presumably, βCC-induced increase in glutamate, glycine, and aspartate accumulation supports increased chlorophyll content in βCC-treated plants. Another vital role of aspartate is to transfer the reduction equivalents from the glycolytic pathway to the mitochondria for ATP generation via the malate–aspartate shuttle [54]. Recent studies showed that mitochondrial components of the malate–aspartate NADH shuttle act as a longevity factor that induces the extension of lifespan in yeast. Therefore, presumably by enhancing the malate–aspartate shuttle, stress-induced excess reducing powers are ameliorated in mitochondria that ultimately helps plant survival during stress. Moreover, the accumulation of aspartate is closely related to stress acclimation. For example, aspartate concentration was increased significantly in drought-exposed *Brassica napus* plants [55]. It is known that out of the eight essential amino acids, four amino acids, namely methionine, threonine, lysine, and isoleucine, are produced from aspartate [56]. An increase in aspartate after βCC treatment is translated into an increase in the levels of homoserine (a common intermediate of methionine, threonine, and lysine), and specifically threonine, but not methionine, lysine, and isoleucine. βCC also induced the accumulation of leucine. An increase in threonine and leucine may be attributed to the critical components of serine/threonine protein kinases [57] and leucine-rich repeat (LRR) proteins [58], respectively, and facilitates the perception of stress signals and protein–protein interactions. An increase in the transcripts of serine/threonine protein kinases and LRR proteins after βCC application [25] suggests the same.

Abiotic stresses, such as drought and salinity, cause osmotic stress in the plant. To maintain osmolarity, plants produce compatible solutes to maintain cell turgor. These non-toxic compounds fall into three categories, namely, amino acids, onium compounds, and sugars/polyols [59]. Proline is one of the significant osmolytes; however, we found proline levels remained unaltered until three days after βCC treatment. Our previous study showed that proline significantly increased after 21 days of βCC treatment. Therefore proline may be upregulated after three days of βCC treatment. However, homoserine is a non-protein amino acid, and increased levels of homoserine can be attributed to the production of the homoserine betain, a known osmolyte in salt stress [60]. Mannose is another important metabolite that also works as an osmolyte and, in addition, enhances antioxidant metabolism and reduces chlorophyll degradation [61].

Accumulation of phytophenols is significantly increased after βCC treatment. They contribute to plant color and protect plants from oxidative stress, pathogen infestation, and herbivore attack [62]. Phytophenols are biosynthesized utilizing amino acids as the precursors; however, a few phenolic compounds are also derived from the shikimic acid pathway [63]. Coumaric acid is one of the phytophenols that is biosynthesized through the shikimate pathway [64] and can be converted into phenolic acids [65,66]. Therefore, an increase in the levels of coumaric acid and its phenolic acids, namely, ferulic acid and its isomer isoferulic acid, suggest a role of βCC in the production of defense metabolites against abiotic and biotic stress. This view is further strengthened by the commonly upregulated defense metabolites in βCC- and simulated-herbivory-treated samples. Interestingly, α-tomatine, a glycoalkaloid specifically present in tomato that deters insect herbivores, is significantly greater in βCC-treated plants than simulated-herbivory-treated plants. Similar trends were evident in the accumulation of quinic acid. Together, this suggests that βCC can upregulate defense metabolites that prime tomato plants against multiple stresses. However, a few metabolites, namely cinnamic acid, nicotinic acid, linoleic acid, solasodin, and solasonin, that are related to plant defense, were downregulated significantly. A high turnover of cinnamic acid to coumaric acid and rutin probably restricts its accumulation. Similarly, the allocation of aspartate towards homoserine production can limit aspartate allocation towards nicotinic acid. Downregulation of linoleic acid after βCC treatment is surprising, as linoleic acid levels are known to be increased within an hour of herbivory and pathogen attack. After being attacked by herbivores, linoleic acid is liberated from cell membranes and is converted to jasmonate, which regulates transcription of the defense genes [67]. In our previous transcriptomic study, we did not find upregulation of any genes from the jasmonate biosynthetic cascade [25]. In the current study, we could not detect jasmonic acids or their derivatives; however, the levels of α-tomatine, a jasmonate-dependent glycoalkaloid [68], increased. Together, these suggest that βCC operates in a jasmonate-independent way in tomato plants. The other glycoalkaloids, solasodine, and solasonine were downregulated after BCC treatment. As α-tomatine is the major glycoalkaloid in tomato plants, downregulation of others may be cost-effective.

## 5. Conclusions

In the current study, we found that exogenous βCC elicits changes in the metabolome of tomato plants. Interestingly, these changes were more significant at early time points after βCC treatment than later ones. βCC mainly regulates amino acid and phytophenol metabolism. Interestingly, βCC-treated plants precisely upregulated metabolites having a role in improving both growth and defense; moreover, regulation of amino acid and phytophenol metabolism at different times optimized the growth of tomato plants. Therefore, βCC is a promising molecule for inducing resilience against biotic and abiotic stress. In general, most research has focused on the effect of priming molecules on plant phenotypic changes. However, our study sought to reveal the molecular changes that contribute to understanding the molecular mechanisms underlying priming.

## Data Availability

The data presented in this study are available in the main text and supplementary materials.

## Supplementary Materials

The following supporting information can be downloaded at: https://www.mdpi.com/article/10.3390/metabo13030329/s1, Figure S1: Representative base peak chromatogram (BPC) from LC-MS analysis of control, βCC-, and simulated-herbivory-treated samples; Figure S2: Fold-change analysis of the samples from early time points (0–240 min) after βCC treatment compared to control plants; Figure S3: Fold-change analysis of the samples from 72 h after βCC treated plants compared to control plants. Table S1: Compounds identified from control and βCC-treated tomato plants by LC-MS/MS; Table S2: Statistical analysis of the levels of discriminant metabolites identified early after βCC treatment.
