# Supplementary material for: β-Cyclocitral-Mediated Metabolic Changes Optimize Growth and Defense Responses in Solanum lycopersicum L
Source: Metabolites. 2023 Feb 23;13(3):329. doi: 10.3390/metabo13030329 (PMC10053473; doi:10.3390/metabo13030329)
Supplement: Supplementary file 1 [file metabolites-13-00329-s001.zip › Supplemental Figures.pdf]

(a) Control 0 minutes

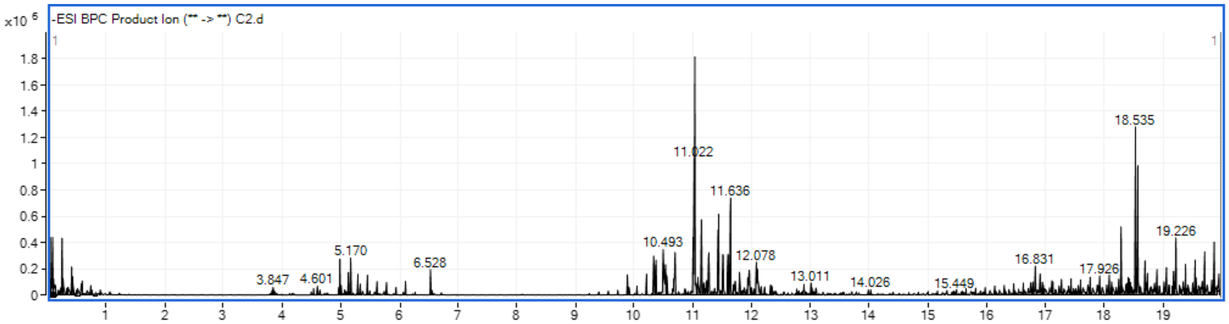

(b) Control 30 minutes

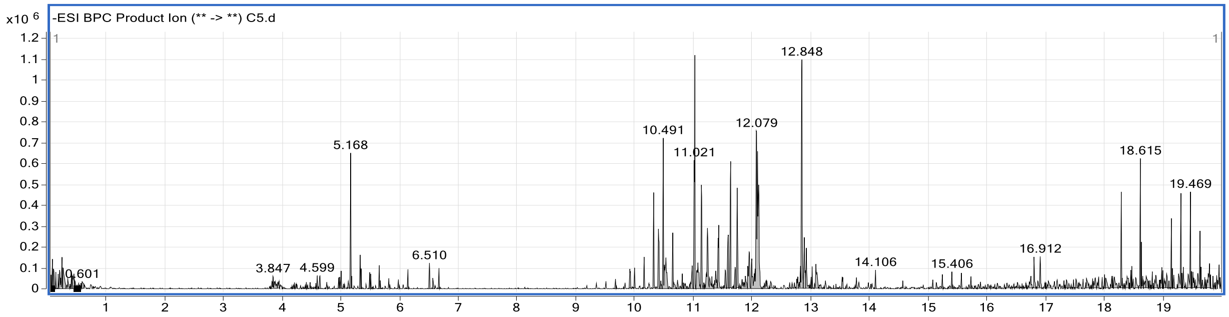

(c) Control 60 minutes

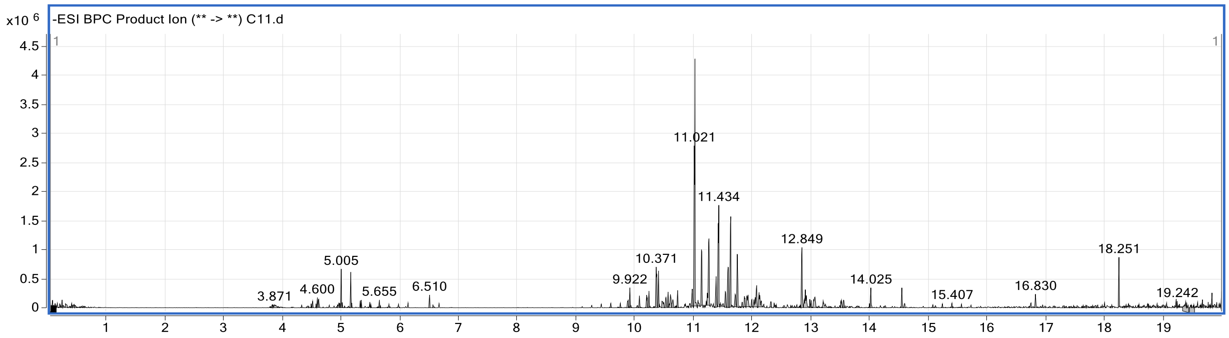

Counts vs. acquisition time (min)

(d) Control 90minutes

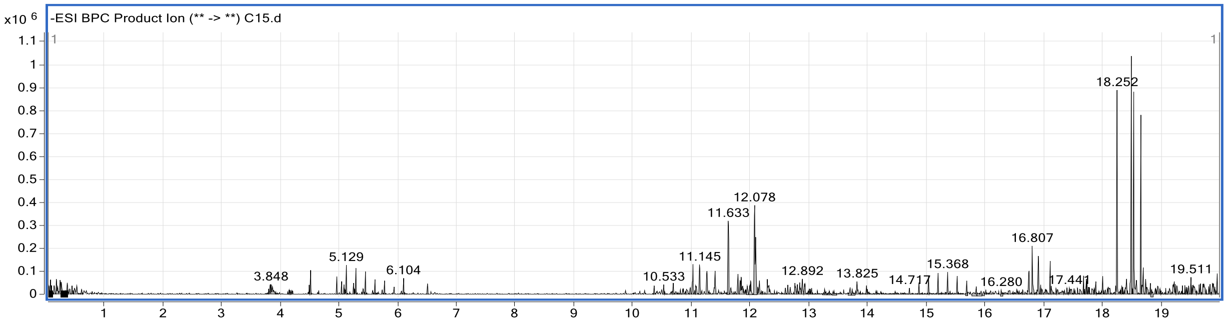

(e) Control 180minutes

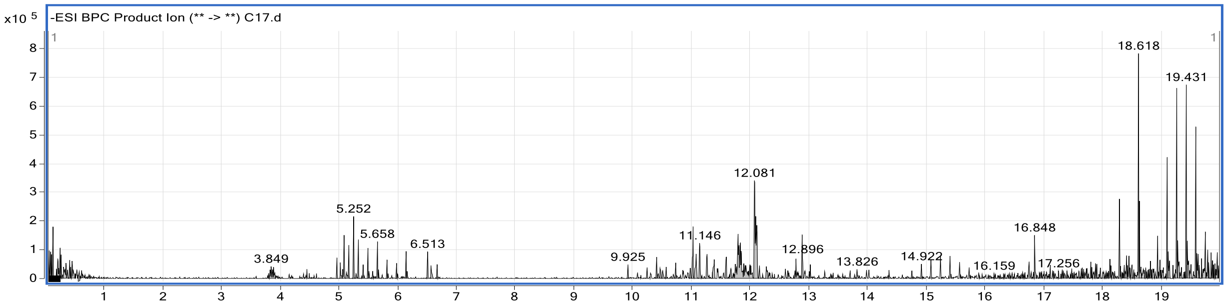

(f) Control 240minutes

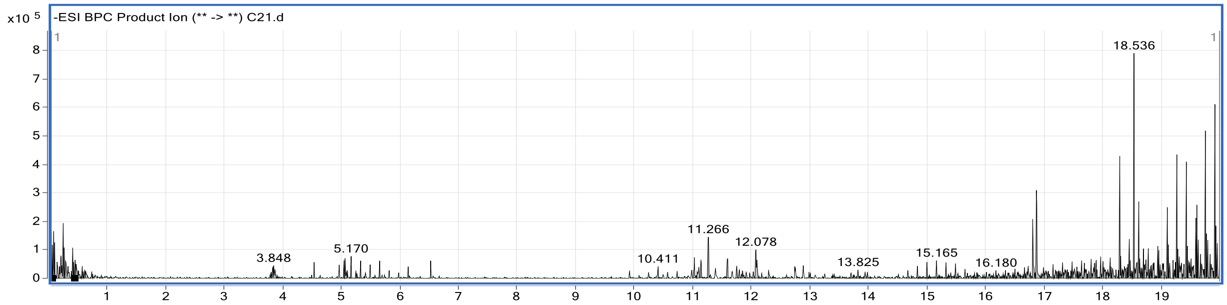

Counts vs. acquisition time (min)

(g) βCC 0 minutes

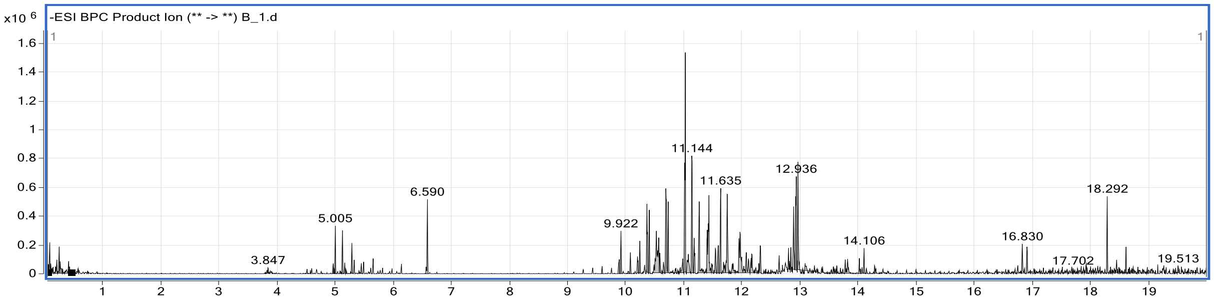

(h) βCC 30 minutes

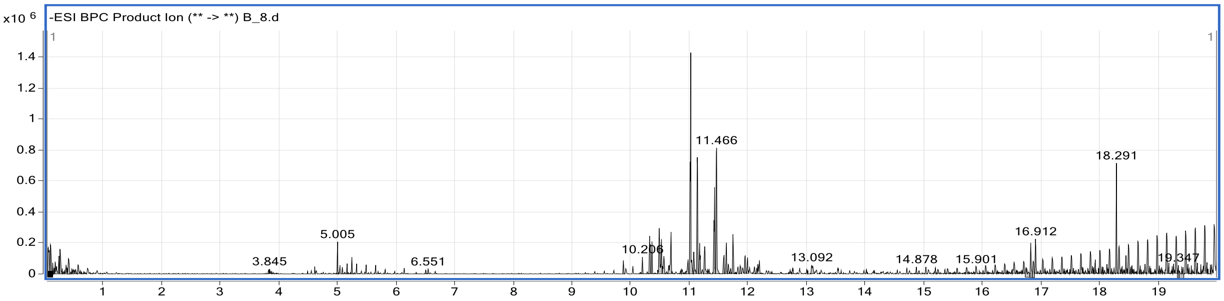

(i) βCC 60 minutes

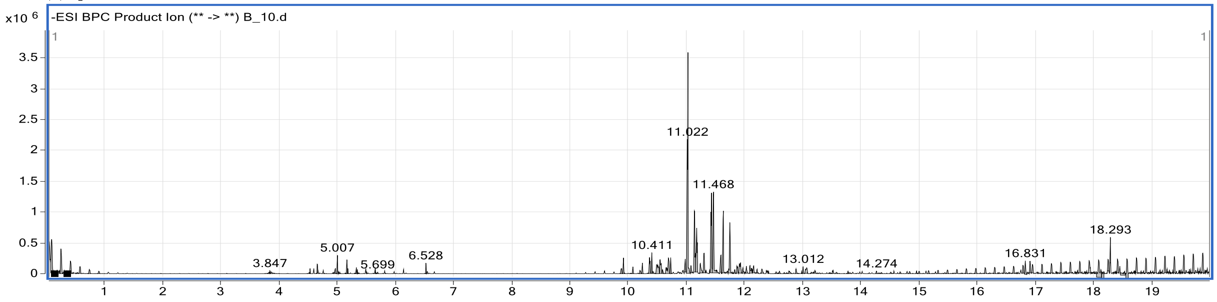

Counts vs. acquisition time (min)

(j) βCC 90minutes

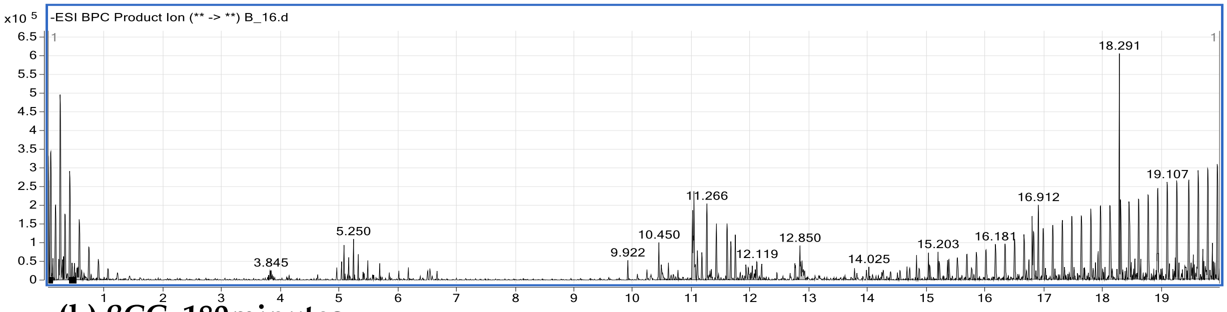

(k) βCC 180minutes

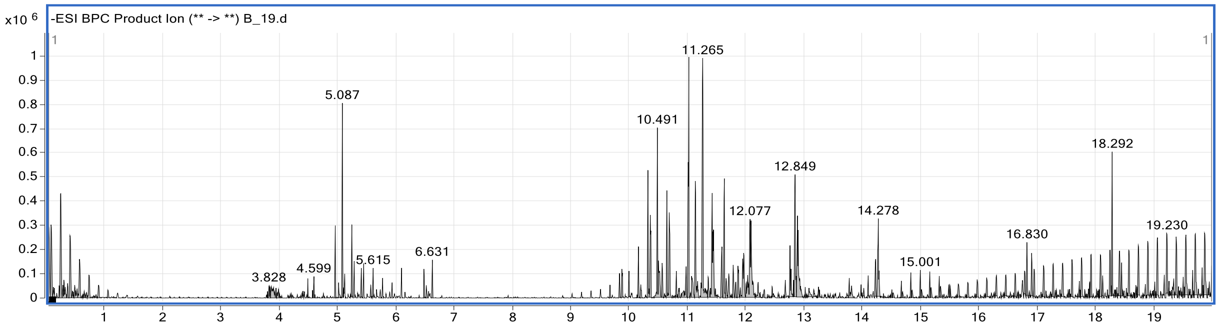

(l) βCC 240minutes

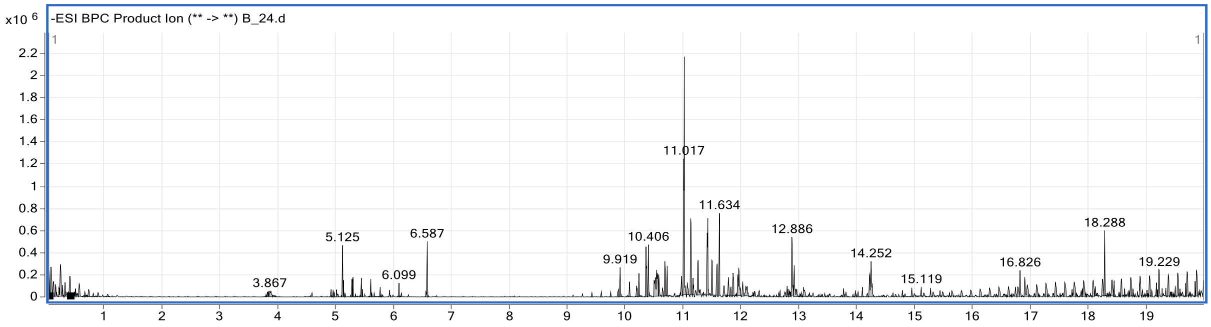

Counts vs. acquisition time (min)

(m) Control 72hours

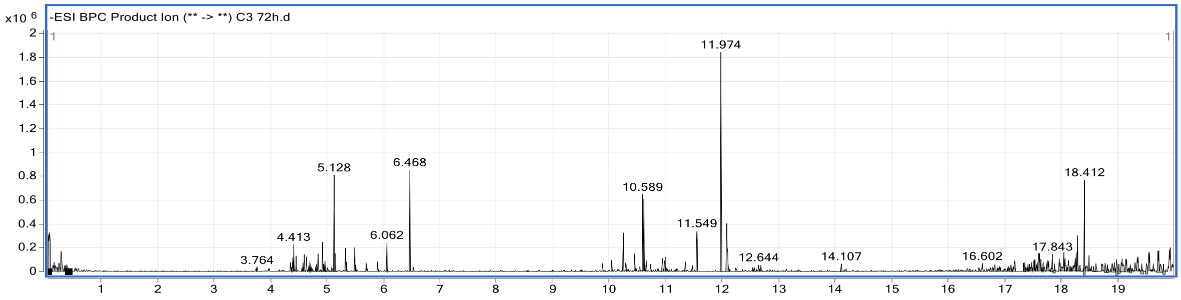

(n) βCC 72 hours

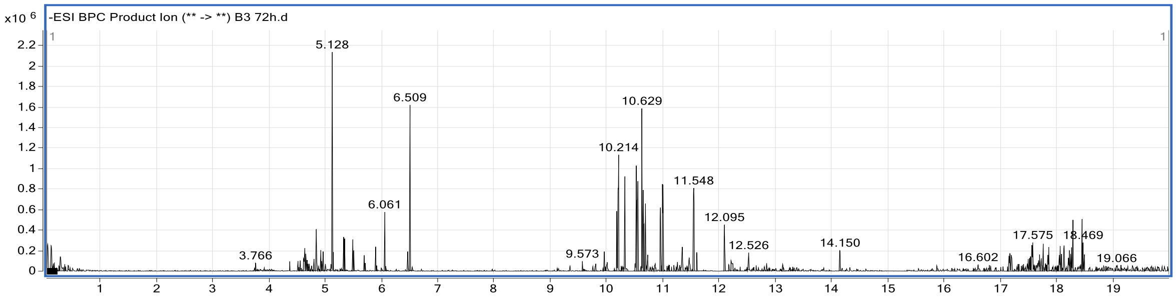

(o) W+OS 72 hours

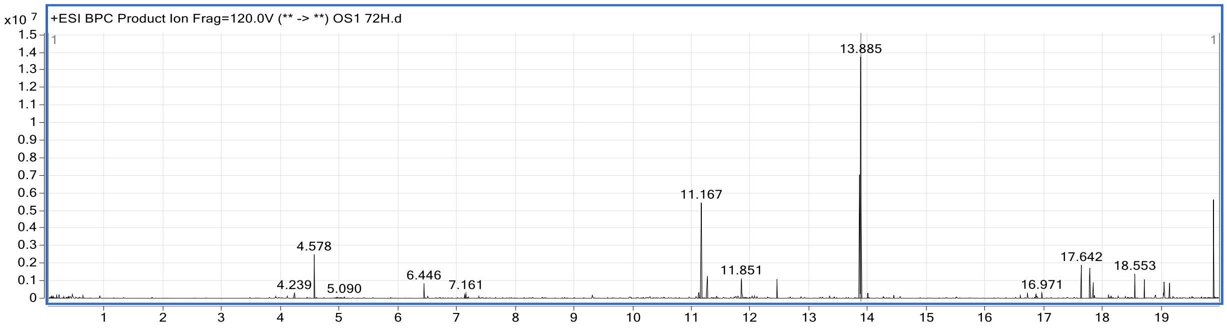

Counts vs. Acquisition Time (min)

Figure S1: Representative base peak chromatogram (BPC) from LC-MS analysis of control, βCC-, and simulated herbivory treated samples.

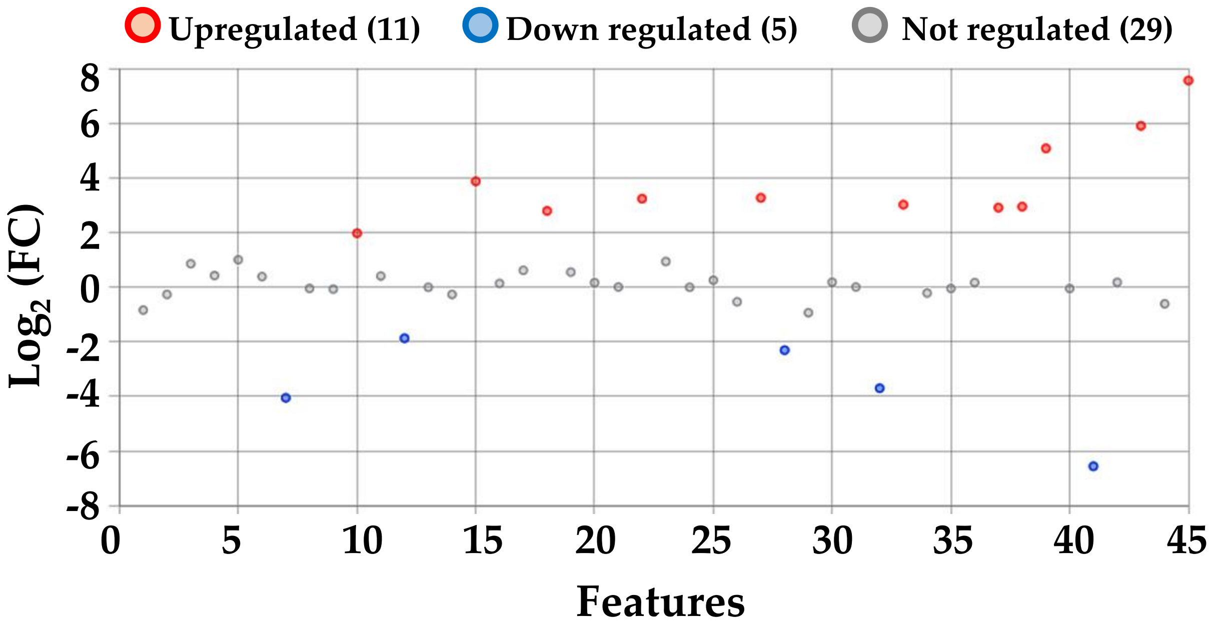

Figure S2: Fold-change analysis of the samples from early time points (0-240 minutes) after βCC treatment as compared to control plants. Normalized peak area from all the samples were subjected to fold-change analysis where -2≥ $\log_2$FC≥ 2.

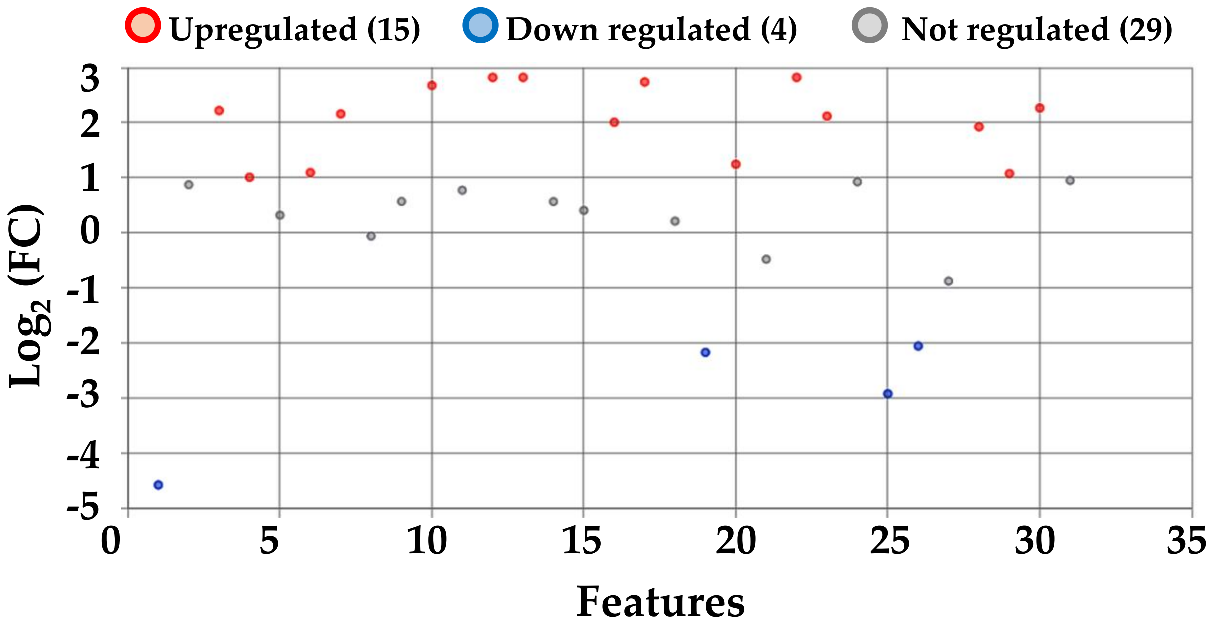

Figure S3: Fold-change analysis of the samples from 72 h after βCC treated plants as compared to control plants. Normalized peak area from all the samples were subjected to fold-change analysis where -2≥ $\log_2$FC≥ 2.
