# Supplementary material for: β-Cyclocitral-Mediated Metabolic Changes Optimize Growth and Defense Responses in Solanum lycopersicum L
Source: Metabolites. 2023 Feb 23;13(3):329. doi: 10.3390/metabo13030329 (PMC10053473; doi:10.3390/metabo13030329)
Supplement: Supplementary file 1 [file metabolites-13-00329-s001.zip › Supplemental Tables.pdf]

Table S1: Compounds identified from control and βCC treated tomato plants by LC-MS/MS. A total of 57 compounds were identified based on the fragment ion spectra in both positive and negative ionization modes. 1-32 compounds are present in positive mode, and 33-57 in negative mode. RT, retention time; #, metabolites identified by matching the molecular and daughter ions from the predicted spectra from HMDB.

Sr. No.	Molecular formula	Abundance (%)	Adducts	Mass	m/z	RT	Score	Compound Name
1	$C_{11}H_{10}O_5$	149(100), 160(56.02), 159(0.46)	(M+H)+	222.0528	223	10.696	79.18	2-Succinylbenzoate
2	$C_{16}H_{18}O_9$	192.05(100), 191.05(41.23), 173.04(5.06)	(M+H)+	354.0951	355.08	10.603	84.08	Chlorogenic acid
3	$C_9H_8O_2$	119.04(78.23), 117.03(54.87), 103.05(23.78)	(M+H)+	148.0524	149.04	14.911	89.04	Cinnamic acid
4	$C_6H_8O_7$	191(100), 175(36.14), 170(18.03)	(M+H)+	192.027	193.0343	6.582	96.46	Citric acid
5	$C_6H_6O_6$	142.99(82.09), 112(26.78), 99.007(1.62)	(M+H)+	174.0164	175.0084	6.54	93.14	Dehydroascorbic acid
6	$C_{50}H_{81}NO_{21}$	1032.541(100),1030.522(72.19), 898.485(25.63)	(M+H)+	1031.53	1076.532	11.903	86018	Dehydrotomatine
7	$C_8H_{11}NO_2$	154.086(100), 154(89.06), 153(38.74)	(M+H)+	153.079	154.0863	4.917	76.02	Dopamine
8	$C_6H_{12}O_6$	163.06(92.64), 145.04(63.74), 127.039(9.34)	(M+Na)+	180.0634	198.0972	4.509	95.59	Galactose
9	$C_4H_9NO_2$	102.07(100), 87.04(6.47), 86.06(2.58)	(M+H)+	103.0633	104.07	4.427	87.57	γ amino butyric acid
10	$C_6H_{12}O_6$	118.9(36.4), 113(13.98), 89(0.64)	(M+H)+	180.0634	179	4.549	91.68	Glucose
11	$C_6H_{13}O_9P$	199(78.02), 138.979(37.51), 96.96 (2.78)	(M+H)+	260.0297	261.022	4.308	88.57	Glucose-6-phosphate
12	$C_5H_9NO_4$	130.05(82.63), 102.07(46.32), 85.047(2.78)	(M+H)+	147.0532	148.06	4.438	86.84	Glutamate
13	$C_5H_{10}N_2O_3$	169.05(100), 130.04(25.33), 101.07(17.69)	(M+H)+	146.0691	147.06	4.346	85.26	Glutamine
14	$C_4H_9NO_3$	119.99(100), 102.04(65.42), 74.125(3.74)	(M+H)+	119.0582	120.065	7.392	80.85	Homoserine
15	$C_{18}H_{32}O_2$	281(100), 280(80.14), 265(23.87)	(M+H)+	280.2402	281.247	19.73	75.37	Linoleic acid
16	$C_6H_{14}N_2O_2$	145.1(100), 130.08(3.61), 119.08(076)	(M+H)+	146.1055	147.11	4.023	78	Lysine
17	$C_{52}H_{85}NO_{23}$	1092(100), 1013(56.13), 383(17.85)	(M+H)+	1091.551	1092	4.143	18.06	Lycoperoside A

Sr. No.	Molecular formula	Abundance (%)	Adducts	Mass	m/z	RT	Score	Compound Name
18	$C_4H_6O_5$	135(100), 117(20.02), 89(16.73)	(M+H)+	134.0215	135.0142	5.687	96.78	Malic acid[#]
19	$C_6H_{12}O_6$	180.19(100), 162(81.02), 85.098(24.87)	(M+H)+	180.0634	181.05	4.55	95.33	Mannose
20	$C_5H_9NO_4$	148.06(100), 106.04(43.12), 88.039(2.54)	(M+H)+	147.0532	148.06	4.43	80.7	O-acetylserine
21	$C_9H_{11}NO_2$	165.05(100), 130.96(37.52), 119.96(2.74)	(M+H)+	165.079	166.08	10.037	92.53	Phenylalanine
22	$C_3H_4O_3$	41(100), 43(33.33), 42(8.41)	(M+HCOO) (M+CH3COO)	88.06	89.0088	5.035	96.73	Pyruvic acid
23	$C_7H_{12}O_6$	191.3(100), 126.08(23.45), 156.9(10.87)	(M+H)+	192.0634	193.0707	4.67	98.6	Quinic acid[#]
24	$C_{27}H_{30}O_{16}$	611(100), 610(77.33), 303.04(14.52)	(M+H)+	610.1534	611.16	11.661	81.92	Rutin
25	$C_{45}H_{73}NO_{16}$	884.5(100), 413.33(50.14), 412.33(49.86)	(M+H)+ (M+Na)+	883.4929	884	11.47	85.25	Solasonin
26	$C_3H_7NO_3$	105.037(100), 88.016(41.23), 60.0154(21.87)	(M+H)+	105.0426	106.049	4.308	78.14	Serine
27	$C_4H_6O_4$	116.955(92.14),99.016(26.74), 73.114(10.05)	(M+H)+ (M+CH3COO)	118.0266	119.019	5.484	92.91	Succinic acid[#]
28	$C_{12}H_{22}O_{11}$	324.92(100), 162.96(47.31), 144.94(8.66)	(M+H)+ (M+HCOO)	342.1162	365.1054	4.509	91	Sucrose
29	$C_4H_9NO_3$	118(100), 102.05(32.47), 84.04(1.62)	(M+H)+	119.0582	120.06	4.346	96.23	Threonine
30	$C_{50}H_{83}NO_{21}$	1034.55(100), 1017.54(80.11) 1016.54(73.55)	(M+H)+	1033.546	1034.553	12.108	75.96	Tomatine
31	$C_{15}H_{24}N_2O_{17}P_2$	566.777(100),565.053(23.84), 542.85(1.64)	(M+H)+	566.055	567.047	4.1	79.51	UDP- Glucose
32	$C_5H_{11}NO_2$	117.99(100), 72.146(20.81), 73.08(2.64)	(M+H)+	117.079	118.0862	6.135	79.91	Valine
33	$C_6H_4N_2O_5$	153(100), 123(2.6), 137.01(0.52)	(M-H)-	184.012	183	18.523	76.02	2,4-Dinitrophenol
34	$C_3H_7O_7P$	166(100), 96.92(25.12), 86.98(2.5)	(M-H)-	185.9929	184.99	4.368	92.98	3-Phosphoglyceric acid
35	$C_6H_{14}N_4O_2$	158(100), 157(8.62), 116(5.37)	(M-H)-	174.1117	173.11	4.104	98.74	Arginine
36	$C_6H_8O_6$	147(100), 113.023(27.63), 87.007(5.21)	(M-H)-	176.0321	175.0241	4.351	92.7	Ascorbic acid
37	$C_4H_7NO_4$	132.04(100), 116.03(34.78), 88.03(9.37)	(M-H)-	133.0375	132.044	4.346	81.26	Aspartate

Sr. No.	Molecular formula	Abundance (%)	Adducts	Mass	m/z	RT	Score	Compound Name
38	$C_7H_6O_2$	123.44(100), 105.045(26.87), 95.049(16.32)	(M-H)-	122.0368	121.0442	13.444	85.43	Benzoic acid
39	$C_9H_8O_4$	163.03(100), 145.02(34.12), 135.04(29.64)	(M-HCOO)-	180.0423	179.04	10.608	85.38	Caffeic acid
40	$C_5H_8O_5$	147.028(100), 129.018(24.85), 87(11.84)	(M-H)- (M+CH3COO)-	148.0372	147.028	7.72	92.8	Citramalic acid
41	$C_9H_8O_3$	163(100), 146(52.63), 123(21.79)	(M-H)-	164.0473	163.0546	13.36	84.85	Coumaric acid
42	$C_{10}H_{10}O_4$	178.026(100), 175.04(84.25), 149.059(52.17)	(M-H)- (M+HCOO)-	194.0579	193.05	14.911	87.68	Ferulic acid
43	$C_4H_4O_4$	115(100), 114.9(8.15), 113.173(0.67)	(M-H)- (M+HCOO)-	116.011	115.0024	5.036	85.63	Fumaric acid
44	$C_6H_{10}O_7$	175.1(100), 131.1(24.81), 113.1(10.67)	(M-H)- (M+CH3COO)-	194.0427	193.035	4.467	92.23	Glucuronic acid
45	$C_5H_8O_4$	130.936(100), 112.97(54.97), 87.075(22.56)	(M+HCOO) (M+CH3COO)-	132.0423	131.03	4.673	99.02	Glutaric acid
46	$C_6H_9N_3O_2$	154.903(100), 136.9(63.79), 135.9(14.23)	(M-H)-	155.0695	154.0622	4.346	85.13	Histidine
47	$C_5H_9NO_3$	130.073(100), 132.066(96.42), 114.055(20.14)	(M-H)-	131.0582	130.0655	7.353	80.85	Hydroxyproline
48	$C_{10}H_{10}O_4$	193.16(100), 178.153(54.36), 134.183(8.74)	(M-H)-	194.0579	193.0506	14.993	84.28	Isoferulic acid
49	$C_6H_{13}NO_2$	130.1(100), 129.3(23.64), 86.09(2.69)	(M-Na)-	131.0946	130.1	4.998	75.23	Isoleucine
50	$C_5H_6O_4$	129(100), 113(23.14), 99(0.36)	(M-HCOO) (M-CH3COO)-	130.0266	129.0339	4.958	89.8	Itaconic acid
51	$C_6H_{13}NO_2$	130.506(100), 86.087(24.87), 80.049(1.98)	(M-Na)-	131.0946	130.101	7.957	75.12	Leucine
52	$C_6H_5NO_2$	122.05(100), 106.01(6.87), 80(2.14)	(M-H)- (M+HCOO)-	123.032	122.03	15.62	85.89	Nicotinic acid
53	$C_7H_6O_3$	137.02(100), 98.462(76.84), 112.03(49.54)	(M-H)- (M+HCOO)-	138.12	137.026	15.35	96.58	Salicyclic acid
54	$C_{27}H_{43}NO_2$	412.33(100), 396.324(87.21), 157.099(30.77)	(M-H)-	413.3294	412.33	18.689	77.22	Solasodine[#]
55	$C_7H_{10}O_5$	173(100), 157(63.47), 129(21.49)	(M+HCOO) (M+CH3COO)-	174.0528	173.06	11.05	77.55	Shikimic acid
56	$C_{11}H_{12}N_2O_2$	203.89(100), 187.87(54.12), 145.92(8.74)	(M-H)- (M+HCOO)-	204.0899	203.09	10.809	92.89	Tryptophan
57	$C_9H_{11}NO_3$	180.08(100), 165.05(23.74), 136.07(7.64)	(M-H)-	181.0739	180.08	18.64	88.7	Tyrosine

Table S2: Statistical analysis of the levels of discriminant metabolites identified early after βCC treatment. The normalized peak area of the upregulated discriminant metabolites identified after 0, 30, 60, 90, 180, and 240 minutes after βCC treatment was compared with that of control plants. Mean normalized peak area (± SE) was analyzed from four replicate plants by one-way ANOVA and Fisher's LSD *post hoc* test. Significant difference is determined at p≤ 0.05. ND, not detected.

Sr. No.	Metabolite name	F value ($F_{12,39}$)	Mean (±SE); *p*- value 0 min	30 min	60 min	90 min	180 min	240 min
**Upregulated metabolites**								
1.	Aspartate	2.039	C= 0.045 (±0.003) B= 0.051 (±0.005) 0.767	C= 0.028 (±0.016) B= 0.052 (±0.018) 0.158	C= 0.030 (±0.018) B= 0.070 (±0.009) 0.026	C= 0.014 (±0.005) B= 0.044 (±0.011) 0.087	C= 0.011 (±0.011) B= 0.038 (±0.005) 0.122	C= 0.023 (±0.013) B= 0.046 (±0.015) 0.191
2.	Leucine	9.665	C= ND B= 0.008 (±0.001) 0.0042	C= ND B= 0.010 (±0.002) 0.0005	C= ND B= 0.010 (±0.002) 0.0002	C= ND B= 0.008 (±0.001) 0.004	C= ND B= 0.010 (±0.002) 0.0004	C= ND B= 0.015 (±0.005) <0.0001
3.	Glutamate	2.059	C= 0.137 (±0.047) B= 0.171 (±0.011) 0.457	C= 0.165 (±0.058) B= 0.205 (±0.017) 0.396	C= 0.154 (±0.051) B= 0.233 (±0.023) 0.095	C= 0.107 (±0.018) B= 0.237 (±0.040) 0.010	C= 0.185 (±0.019) B= 0.247 (±0.032) 0.185	C= 0.164 (±0.013) B= 0.254 (±0.014) 0.061
4.	Tryptophan	12.388	C= ND B= 0.103 (±0.017) <0.0001	C= ND B= 0.122 (±0.012) <0.0001	C= ND B= 0.077 (±0.030) 0.0003	C= ND B= 0.080 (±0.008) 0.0002	C= ND B= 0.067 (±0.020) 0.0014	C= ND B= 0.081 (±0.021) 0.0002
5.	Homoserine	49.842	C= ND B= 0.034 (±0.004) <0.0001	C= ND B= 0.028 (±0.003) <0.0001	C= ND B= 0.029 (±0.003) <0.0001	C= ND B= 0.039 (±0.001) <0.0001	C= ND B= 0.045 (±0.006) <0.0001	C= ND B= 0.026 (±0.004) <0.0001
6.	O-acetylserine	45.476	C= ND B= 0.171 (±0.011) <0.0001	C= ND B= 0.205 (±0.017) <0.0001	C= ND B= 0.233 (±0.023) <0.0001	C= ND B= 0.237 (±0.040) <0.0001	C= ND B= 0.247 (±0.032) <0.0001	C= ND B= 0.254 (±0.014) <0.0001
7.	Shikimate	7.444	C= ND B= 0.015 (±0.005) 0.0221	C= ND B= 0.037 (±0.005) <0.0001	C= ND B= 0.015 (±0.006) 0.022	C= ND B= 0.020 (±0.007) 0.022	C= ND B= 0.016 (±0.007) 0.0122	C= ND B= 0.018 (±0.007) 0.005
8.	Coumaric acid	4.166	C= 0.002 (±0.001) B= 0.009 (±0.005) 0.186	C= 0.005 (±0.002) B= 0.011 (±0.006) 0.243	C= 0.005 (±0.003) B= 0.017 (±0.003) 0.024	C= 0.003 (±0.001) B= 0.001 (±0.001) 0.74	C= 0.002 (±0.001) B= 0.025 (±0.007) <0.0001	C= 0.002 (±0.001) B= 0.11 (±0.005) 0.107
9.	Rutin	36.992	C= ND	C= ND	C= ND	C= ND	C= ND	C= ND

			B= 1.424<br>(±0.135)<br><0.0001	B= 1.656<br>(±0.243)<br><0.0001	B= 2.184<br>(±0.322)<br><0.0001	B= 1.240<br>(±0.322)<br><0.0001	B= 1.045<br>(±0.129)<br><0.0001	B= 1.376<br>(±0.143)<br><0.0001
10.	Salicylic acid	2.783	C= ND<br>B= 0.002<br>(±0.002)<br>0.396	C= ND<br>B= 0.003<br>(±0.001)<br>0.089	C= ND<br>B= 0.003<br>(±0.001)<br>0.132	C= ND<br>B= 0.007<br>(±0.003)<br>0.001	C= ND<br>B= 0.004<br>(±0.001)<br>0.049	C= ND<br>B= 0.004<br>(±0.001)<br>0.057
11.	UDP-glucose	5.999	C= ND<br>B= 2.457<br>(±0.522)<br>0.007	C= ND<br>B= 3.637<br>(±0.833)<br>0.0002	C= ND<br>B= 4.006<br>(±1.756)<br><0.0001	C= ND<br>B= 0.606<br>(±0.195)<br>0.487	C= ND<br>B= 0.662<br>(±0.142)<br>0.448	C= ND<br>B= 1.540<br>(±0.002)<br>0.604
12.	Galactose	16.399	C= ND<br>B= 0.142<br>(±0.009)<br><0.0001	C= ND<br>B= 0.086<br>(±0.020)<br>0.0013	C= ND<br>B= 0.130<br>(±0.024)<br><0.0001	C= ND<br>B= 0.171<br>(±0.040)<br><0.0001	C= ND<br>B= 0.131<br>(±0.014)<br><0.0001	C= ND<br>B= 0.123<br>(±0.029)<br><0.0001
**Downregulated metabolites**								
13.	Cinnamic acid	2.947	C= 0.007<br>(±0.003)<br>B= ND<br>0.256	C= 0.015<br>(±0.004)<br>B= ND<br>0.0218	C= 0.015<br>(±0.007)<br>B= ND<br>0.026	C= 0.011<br>(±0.004)<br>B= ND<br>0.084	C= 0.021<br>(±0.011)<br>B= ND<br>0.0016	C= 0.008<br>(±0.003)<br>B= ND<br>0.22
14.	Linoleic acid	10.045	C= 0.017<br>(±0.002)<br>B= ND<br><0.0001	C= 0.013<br>(±0.001)<br>B= 0.001<br>(±0.001)<br><0.0001	C= 0.006<br>(±0.004)<br>B= 0.005<br>(±0.003)<br>0.533	C= 0.004<br>(±0.002)<br>B= 0.004<br>(±0.002)<br>0.79	C= 0.010<br>(±0.001)<br>B= ND<br>0.0005	C= 0.002<br>(±0.002)<br>B= ND<br>0.532
15.	Nicotinic acid	7.143	C= 0.003<br>(±0.001)<br>B= ND<br>0.019	C= 0.003<br>(±0.001)<br>B= ND<br>0.036	C= 0.001<br>(±0.001)<br>B= ND<br>0.339	C= 0.008<br>(±0.001)<br>B= ND<br><0.0001	C= 0.005<br>(±0.002)<br>B= ND<br>0.0009	C= 0.001<br>(±0.001)<br>B= ND<br>0.437
16.	Sucrose	11.259	C= 0.044<br>(±0.014)<br>B= ND<br>0.017	C= 0.073<br>(±0.006)<br>B= ND<br>0.0002	C= 0.087<br>(±0.013)<br>B= ND<br><0.0001	C= 0.059<br>(±0.019)<br>B= ND<br>0.0019	C= 0.110<br>(±0.025)<br>B= ND<br><0.0001	C= 0.073<br>(±0.022)<br>B= ND<br>0.0002
